# The Grand Challenge: Use of a New Approach in Developing Policies in the Area of Radiation and Health

**DOI:** 10.3389/fpubh.2014.00050

**Published:** 2014-05-21

**Authors:** Dariusz Leszczynski

**Affiliations:** ^1^Department of Biochemistry and Biotechnology, University of Helsinki, Helsinki, Finland

**Keywords:** radiation-related health risks, health and risk communication policies, health policies, ionizing or non-ionizing radiation, science-based health protecting policies

People are continuously exposed to radiation emitted by the natural and man-made sources. Exposures to ionizing and to non-ionizing radiation are known to impact human health. Effects exerted by any type of radiation might be potentiated by co-exposures to other types of radiation as well as by co-exposures to chemicals and other environmental pollutants. Inherent individual sensitivity of persons, governed by genetics and epigenetics, plays a role in development of pathological processes.

In the mind of a layperson, radiation, whether ionizing or non-ionizing, carries often a stigma of a “danger,” and cancer is the dominating fatal health outcome in the laypersons’ consideration. This is not always the correct conclusion. Layperson does not always consider the radiation dose that is of paramount importance for the induction of radiation-related health effects and for assessing and assigning any health risk significance. The mere presence of radiation in the environment is not always sufficient to be considered as dangerous for health.

Radiation exposures are known to induce cancer and genomic instability [for review see (1, 2)]. Besides the role in carcinogenicity, radiation has also an impact on the physiology unrelated to cancer, e.g., radiation-induced atherosclerosis and cardiovascular damage (3, 4) or the induction of cataracts (5).

Scientific knowledge should always be the primary basis of any health and risk communication policies. When evaluating the scientific evidence we need not only to determine what we know but also what we do not know, what important topics were not yet studied and what are the gaps in the scientific knowledge. Only by combining the information on what we know and presenting it in the context of what we do not know, we can reliably evaluate the adequacy of the current scientific basis of the health protecting measures and policies.

In radiation protection, as with any environmental factor, whether naturally occurring or man-made, several types of scientific evidence are needed in order to determine human health risks:

- the possible mechanism how the effect is induced in living organism,
- *in vitro* laboratory studies confirming the existence of biophysical and biochemical mechanisms of the effect,
- animal studies determining the effects of exposures to very high doses of the examined factor as well as life-time exposures to doses resembling the levels of human exposures,
- human volunteer studies, whenever ethically permissible, determining whether the effects observed *in vitro* and in animal studies occur in humans and whether the extent of the effects is sufficient to alter normal human physiology,
- epidemiological evidence of the impact of radiation exposures on the health of human population.

Each type of the evidence has different significance and value for the estimation and proving human health effect and, consequently, in defining health policies. Epidemiological evidence is often considered as the most important, followed by the human volunteer studies and animal experiments. *In vitro* evidence does not directly inform about the possible health impact in humans, but it provides information on the possible mechanism of the effect. Knowing the mechanism of the effect increases the reliability of the evidence gathered in epidemiological, human volunteer, and animal studies. In the ideal situation, all above-listed types of scientific evidence will point into the same direction.

Epidemiological studies are often expected to provide the ultimate answer whether radiation exposure is hazardous to humans. However, discovering and validation of any potential health hazard using epidemiological approach is seriously hampered by the low sensitivity of the epidemiological methodology. The low sensitivity of the epidemiological approach means that it can detect strong manifestations of health effects in human population but it is often insufficient and inadequate to reliably detect the health impact of the weak biological effects that might be induced by the low doses of radiation. To prove the causality link of rare effects or rare diseases, epidemiological approach requires establishing of very large cohorts, which is both difficult to achieve in practice and very costly (6, 7). Hence, epidemiological evidence is often too ambiguous to be reliably interpreted and used in the development of health policy. These are the circumstances where the non-epidemiological scientific evidence becomes of paramount importance for policy makers.

Current science is clear on the health risks of the acute exposures to high doses of ionizing or non-ionizing radiation. However, knowledge of the: (i) delayed effects of radiation exposure, (ii) effects of the chronic exposures to radiation and, especially, (iii) the effects of the exposures to low-dose radiation, is insufficient to reliably guide health policy.

The borderline between high, moderate, and low doses of radiation is not a rigid one and varies somewhat between studies and research groups. Based on measurements of cell damage or death using human lymphocytes, low doses of ionizing radiation have been judged to be 20–40 mSv. Data from epidemiological studies suggest a value of 200 mSv for the upper limit for low-dose exposure. Based on animal studies on induction of solid tumors and leukemia, a value of 0.1 Gy/min has been suggested as a low dose-rate regardless of the total dose (8). Recently, values of 100 mSv (9) and 0.5 Gy (10) have been considered as low doses. For UV radiation (180–400 nm), the low doses are considered to be the doses (per square meter) below the exposure limit of 30 J/m^2^ (effective spectrally weighted) set by the International Commission on Non-Ionizing Radiation Protection (ICNIRP) (11). For radio-frequency electromagnetic fields (RF-EMF) of the low values may be considered to be the values below the absorbed “dose-rate” per kilogram of 2 W/kg (for 100 kHz–10 GHz) as specified by the present ICNIRP Guidelines (12).

Some examples of the ongoing scientific controversies about radiation-involvement in the induction of human health effects are, e.g., delayed non-cancer effects of high doses of ionizing radiation (13, 14), bystander effects of low-dose ionizing radiation (15), children leukemia in the vicinity of nuclear power plants (9) or electric power lines (16), and health effects of wireless communication devices (17–19). Later on these scientific controversies make it difficult to reliably determine human health risk and to set reliable health policies.

Elucidation and prediction of all possible biological and health-related effects of radiation are currently hampered by the insufficient knowledge of the molecular-level effects. The low-dose effects are small and, therefore, difficult to discover and assess their impact on normal human physiology. The limiting factor is the lack of the knowledge of the molecular targets absorbing radiation and the mechanisms of triggering the molecular chain of events leading to observed biological effects. The acute biological effects of the high doses of radiation are known. However, predicting the delayed responses – occurring often many years after the initial exposure – is difficult because of the lack of the knowledge of the diversity of the molecular signaling pathways activated by the initial exposure.

The comprehensive discovery of the molecular targets and signaling pathways activated by the radiation exposures is now within the reach. The modern high-throughput screening techniques (HTSTs) allow global evaluation of the changes in cellular genome, transcriptome, proteome, and metabolome. The use of the HTSTs in studying biological effects of radiation exposures will reveal biological effects not possible to predict on the basis of the currently available knowledge (20).

High-throughput screening techniques are already widely used in clinical research in the search of biomarkers of diseases (21–26) and in environmental toxicology (27–30). Further on, their use has been shown to provide new insights into the biological mechanisms of, e.g., aerial pollutant – benzene (31) and water pollutant – arsenic (32); re-examining the presently known effects of radiation by using the HTST will lead to a broader and more comprehensive picture of the radiation effects.

With the help of HTSTs, a large amount of information on the global changes in physiology of living organisms can be obtained rapidly and efficiently, when compared with more traditional non-HTST approaches. Combining the information obtained with HTST on the responses of human genome, transcriptome, proteome, and metabolome allow gaining new insights into the normal and pathological physiology of cells, tissues, and organisms. At this point, because the HTST-analyses of the vast numbers of genes, transcripts, proteins, and metabolites may produce variety of false positive discoveries (33, 34), screening with HTSTs has to be considered as just the beginning of the discovery process. Therefore, the validity of the changes detected by using HTST and hypotheses built on these discoveries need to be confirmed by other, non-HTST methods.

Research of biological and health effects of radiation is still waiting for the “information boom” already delivered in other research areas by the use of HTSTs. Search through the scientific literature shows only a very limited number of studies using HTSTs in examining the effects of radiation, as seen in the recent review of the studies using systems biology approach (35) and proteomics methods (36–38).

Introduction of any regulatory measures needs to be rooted in the science, and any changes should stem from the *new* scientific information. “Radiation and Health,” a specialty of “Frontiers in Public Health,” is aimed at the scientific community and at the decision makers interested in health effects caused by exposures to radiation. The aim of “Frontiers in Public Health – Radiation and Health” is to provide a comprehensive overview of the science that form the basis for health policy decisions and used to develop new health policies. This task will be achieved through publication of scientific studies from the diverse research areas examining:

- policies to mitigate radiation-related health risks,
- risk estimation of radiation exposures of individuals as well as of human population as a whole,
- new, science-based, approaches of risk communication about the dangers of radiation exposures that will emphasize reliable and open information to avoid triggering of unfounded scare,
- the impact caused by radiation exposure on both physiological and psychological aspects of human health examined by epidemiological approach in population,
- effects of radiation exposures on human physiology by biomedical approaches,
- animal radiation toxicology studies primarily designed to assist in estimation of human health risk caused by acute and chronic radiation exposures,
- *in vitro* laboratory studies examining biophysical and biochemical mechanisms of the physiological effects of radiation exposures (*in vivo* and *ex vivo*) on human tissues and cells,
- “omics” studies that examine globally effects of radiation on humans and animals using high-throughput methods of genomics, transcriptomics, proteomics, and metabolomics; the “omics” approach will help to comprehensively discover signaling pathways and molecules targeted by radiation exposures and to develop new and more precise hypotheses about known as well as yet undiscovered physiological effects of radiation.

The Grand Challenge for “Frontiers in Public Health – Radiation and Health” is to broadly report on the implementation of the modern research methods of genomics, transcriptomics, proteomics, and metabolomics to study effects of radiation exposures on living matter. The comprehensive knowledge of the diverse molecular basis of the origin of the biological effects will help to develop better ways and means for predicting all potentially possible detrimental effects of radiation exposures, both the cancer-related and non-cancer outcomes. The Grand Challenge for future radiation research is to provide new molecular-level scientific information, by examining new research areas and revisiting the old ones. This global information will be then available for the public policy makers, allowing them to develop new and better, firmly science-based health protecting policies.

## Conflict of Interest Statement

The author declares that the research was conducted in the absence of any commercial or financial relationships that could be construed as a potential conflict of interest.
